# A systematic review of the characteristics and validity of monitoring technologies to assess Parkinson’s disease

**DOI:** 10.1186/s12984-016-0136-7

**Published:** 2016-03-12

**Authors:** Catarina Godinho, Josefa Domingos, Guilherme Cunha, Ana T. Santos, Ricardo M. Fernandes, Daisy Abreu, Nilza Gonçalves, Helen Matthews, Tom Isaacs, Joy Duffen, Ahmed Al-Jawad, Frank Larsen, Artur Serrano, Peter Weber, Andrea Thoms, Stefan Sollinger, Holm Graessner, Walter Maetzler, Joaquim J. Ferreira

**Affiliations:** Instituto de Medicina Molecular, Faculdade de Medicina, Universidade de Lisboa, Avenida Professor Egas Moniz, 1649-028 Lisboa, Portugal; Laboratory of Clinical Pharmacology and Therapeutics, Faculty of Medicine, University of Lisbon, Lisbon, Portugal; Center for Interdisciplinary Research Egas Moniz (CiiEM), Instituto Superior de Ciências da Saúde Egas Moniz, Monte de Caparica, Portugal; CNS-Campus Neurológico Sénior, Torres Vedras, Portugal; The Cure Parkinson’s Trust, London, UK; HSG-IMIT, Villingen-Schwenningen, Germany; Norwegian Centre for Telemedicine, Tromso, Norway; Hasomed GmbH, Magdeburg, Germany; AbilityNet, London, UK; Institute for Medical Genetics and Applied Genomics, University of Tuebingen, Tuebingen, Germany; Department of Neurodegeneration, Hertie Institute for Clinical Brain Research, Center of Neurology, University of Tuebingen, Tuebingen, Germany

**Keywords:** Outcomes, Parkinson’s disease, Quantitative assessment, Wearable devices, Monitoring technologies

## Abstract

**Background:**

There is growing interest in having objective assessment of health-related outcomes using technology-based devices that provide unbiased measurements which can be used in clinical practice and scientific research. Many studies have investigated the clinical manifestations of Parkinson’s disease using such devices. However, clinimetric properties and clinical validation vary among the different devices.

**Methods:**

Given such heterogeneity, we sought to perform a systematic review in order to (i) list, (ii) compare and (iii) classify technological-based devices used to measure motor function in individuals with Parkinson's disease into three groups, namely wearable, non-wearable and hybrid devices. A systematic literature search of the PubMed database resulted in the inclusion of 168 studies. These studies were grouped based on the type of device used. For each device we reviewed availability, use, reliability, validity, and sensitivity to change. The devices were then classified as (i) ‘recommended’, (ii) ‘suggested’ or (iii) ‘listed’ based on the following criteria: (1) used in the assessment of Parkinson’s disease (yes/no), (2) used in published studies by people other than the developers (yes/no), and (3) successful clinimetric testing (yes/no).

**Results:**

Seventy-three devices were identified, 22 were wearable, 38 were non-wearable, and 13 were hybrid devices. In accordance with our classification method, 9 devices were ‘recommended’, 34 devices were ‘suggested’, and 30 devices were classified as ‘listed’. Within the wearable devices group, the Mobility Lab sensors from Ambulatory Parkinson’s Disease Monitoring (APDM), Physilog®, StepWatch 3, TriTrac RT3 Triaxial accelerometer, McRoberts DynaPort, and Axivity (AX3) were classified as ‘recommended’. Within the non-wearable devices group, the Nintendo Wii Balance Board and GAITRite® gait analysis system were classified as ‘recommended’. Within the hybrid devices group only the Kinesia® system was classified as ‘recommended’.

**Electronic supplementary material:**

The online version of this article (doi:10.1186/s12984-016-0136-7) contains supplementary material, which is available to authorized users.

## Background

The introduction of technology-based devices in medical care has been considered a cutting edge advance in modern medicine. There is a growing interest in having objective assessment of health-related outcomes using these devices which provide unbiased measurements and can be used in both daily clinical practice and scientific research. Several factors facilitate this interest, namely the ubiquitous nature of technology in the home-environment, the growing access to high-speed Internet connections and the rising computer literacy of the general population.

Additionally, technology-based devices may simplify patient participation and data management in clinical trials. They may ultimately enable long-term follow-up of previously established outcomes, with the ability to detect subtle changes that would otherwise go unnoticed. Such devices have been used in a variety of illnesses, such as breast cancer, chronic obstructive pulmonary disease, osteoarthritis, stroke, and Parkinson’s disease (PD).

Many studies have investigated the clinical manifestations of PD using technology-based devices. However, clinimetric properties and clinical validation vary among the different devices. Given such heterogeneity, this study proposes a systematic review of current literature aiming to (i) list, (ii) compare and (iii) classify technology-based devices that objectively measure PD motor symptoms.

## Methods

Medline on PubMed was searched to identify the relevant papers for all listed publications published up to November 2015. The following MeSH terms were used, Parkinson’s disease OR symptoms AND devices OR monitoring OR assessment, combined with a sensitive filter to complete the literature search (namely, publication date, article type of study, text availability, languages, and species). Additionally, citation and reference reviews were conducted manually to identify any additional suitable studies. Two researchers performed the bibliographic referencing (JD and CG). Studies were included if they met the following criteria: (1) devices having been used with people with PD; and (2) devices that produced objective, quantifiable outcomes. In case of discrepancy, a common ground solution was found. Studies were excluded if (1) Non PD population, (2) the outcomes were measured using only subjective scales or questionnaires; (3) devices were used as a complementary diagnostic work-up (e.g., datscan), (4) devices used for treatment purposes and (5) the articles were published in languages other than English.

Based on the portability and type of technology used, the studies were classified into three groups, namely (i) wearable, (ii) non-wearable and (iii) hybrid. Wearable devices were defined as electronic technology or computers designed to be worn on the body, or embedded into watches, bracelets, clothing, and others. Hybrid devices were defined as the blend of technologies that combined wearable and non-wearable devices. For each device, a search was then conducted for the terms “Parkinson’s disease” and the name of the device looking for original articles related to the development and use of each individual device in Medline.

Data were systematically extracted using a customized table (see Additional file 1: Table S1) based on a Movement Disorder Society template [1, 2], and adapted to the specific needs of this review. This table enabled the evaluation of descriptive features, availability, and outcomes to be measured, as well as clinimetric properties.

Devices were classified in order of recommendation based on the following criteria: (1) having been used in the assessment of PD symptoms and signs; (2) having been used by people other than the developers (Yes/No); and (3) having had successful reported clinimetric testing (Yes/No). The devices were classified as “recommended” if they met three criteria, “suggested” if they met only two of the criteria or “listed” if they met one criterion only [3]. This classification has previously been applied in other reviews of scales and assessment tools in PD [3].

Clinimetric testing was considered successful if a device was shown to be (1) reliable, (2) valid and (3) sensitive to change. Reliability was defined as the degree to which the measurement is free from measurement error [4]. The Intra Class Coefficient (ICC) is the most common reliability parameter for continuous measurements. The ICC is, however, highly dependent on the between subject variation of the measurement within the sample studied and may give highly divergent findings applied to samples chosen in different ways from the same population.

Test-retest reliability is an adequate test to assess intra-rater reliability. Validity is the degree to which an instrument measures the construct it purports to measure [4]. Validity contains the following measurement properties: content validity, construct validity and criterion validity. Sensitivity to change or responsiveness reflects the ability of an instrument to detect change over time [4]. Quality cut-offs of clinimetric properties have been proposed by previous studies [5]. However, they have not been applied to this study due to the diversity of study designs (number of subjects, methods, statistical analysis) that compromises any productive analysis.

## Data synthesis and results

**Fig. 1:**
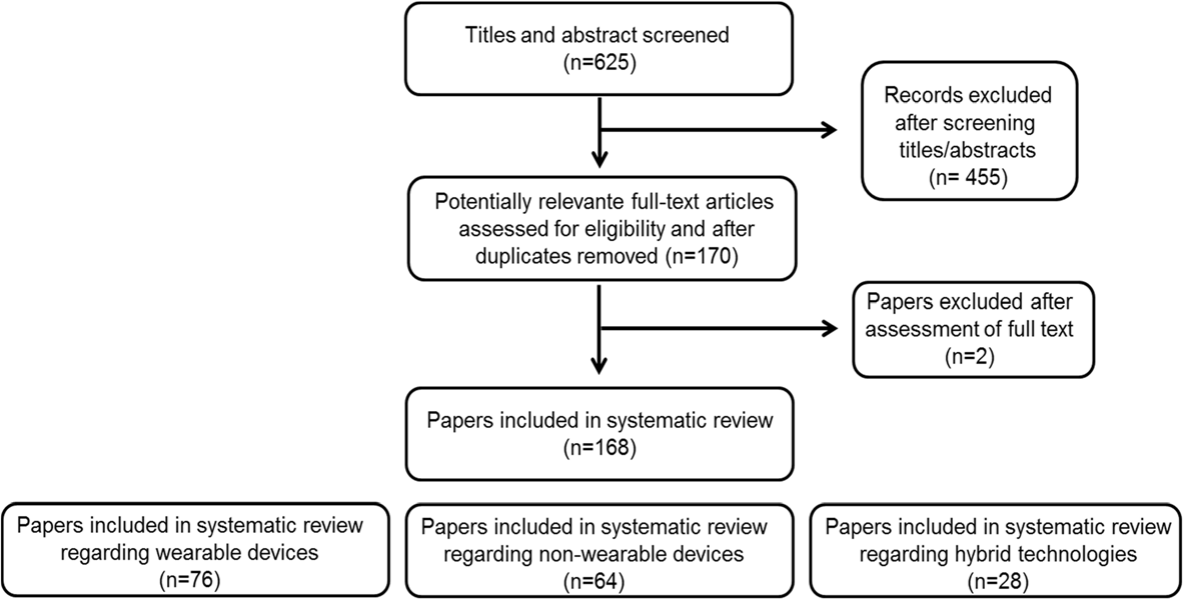
Flow diagram for data extraction

The literature search undertaken yielded 625 records, of which a total of 168 articles were included in full-text format for further evaluation. They were divided in three categories (Fig. 1): (1) “wearable”, (76 articles); (2) “non-wearable”, (64 articles); and (3) “hybrid” (28 articles). A total of 73 devices were identified, of which 22 were from the “wearable” category, 38 from the “non-wearable” category, and 13 from the “hybrid” category. An overview of the devices classified as “recommended” are highlighted in Table 1 and in the main text. All the rest of the devices were only included in a Additional file 1: Table S1.

**Table 1:** Overview of the assessed instruments classified as recommended

Device	Clinical Parameter	External usage	Clinimetric properties		
			Reliability	Validity	Sensitivity to change
Wearable Devices					
Mobility Lab System (APDM)	Bradykinesia Dyskinesia Postural control Gait/FOG ADLs	X	X	X	X
Physilog®	Postural control Gait/FOG Physical Activity Tremor Bradykinesia	X	X	X	X
StepWatch 3 (SAM)	ADLs Gait	X	X	X	X
TriTrac RT3	ADLs	X	X	X	X
McRoberts Dynaport	ADLs Falls/near Falls Gait	X	X	X	X
Axivity (AX3)	Physical Activity/ADLs Falls Gait	X	X	X	X
Non-Wearable Devices					
Wii Balance Board	Postural instability	X	X	X	X
GAITRite®	Bradykinesia (axial)Gait/FOG	X	X	X	X
Hybrid technologies Network Platforms and Telemedicine					
Kinesia™	Tremor	X	X	X	X

(X) Information available

### Wearable devices

#### Mobility Lab system (APDM)

##### Device description and outcomes that can be measured

Ambulatory Parkinson’s Disease Monitoring (APDM) is both the name of the system as well as the company. The analysis software is called the Mobility Lab. The system comes with a Clinical Data Management System (CDMS) called Mobility Exchange.

It is a watch-sized device consisting of up to six sensors (including an accelerometer, gyroscope and magnetometer) and hold a charge for over 16 h. It enables the registration of different outcomes such as, postural sway - ISway (velocity, frequency, distance), lower limb gait (cadence, stride length, stride velocity, gait cycle time), postural transitions (number of steps, duration, step time), upper limb gait and trunk (trunk range of motion) measures.

The software has several modules such as iSway and iTUG (instrumented Timed Up and Go test). The iTUG test enables the assessment of different parameters in all sub-tasks of the test: (1) sit to stand (duration, peak velocity and trunk range of motion); (2) gait (stride length, stride velocity, cadence gait cycle time, and double support) and trunk movements; (3) turning (number of steps, peak velocity and step time); and (4) Turn to sit (peak turn velocity and trunk range of motion).

##### Clinimetric properties

In a study with 12 people with PD and 12 controls the iTUG measures were correlated with the UPDRS-III (Unified Parkinson’s Disease Rating Scale–motor part) (*r* =–0.73, *p* = 0.006). The iTUG test was shown to be sensitive to untreated PD and to potentially detect progression of PD and response to symptomatic treatments [6].

These proprieties were also studied in two separate studies with two groups of subjects. In study I [7] sensitivity and experimental concurrent validity were assessed by testing 13 subjects with early, untreated PD and 12 age-matched control subjects in the laboratory comparing sway from force-plate CoP (center of pressure) and inertial sensors. In study II [8], test-retest reliability and clinical concurrent validity were assessed by testing 17 early-to-moderate treated PD, and 17 age-matched control subjects in a clinical setting, comparing clinical balance tests with sway from inertial sensors [8].

Both CoP and 2D time series acceleration (ACC) measures differentiated sway between controls and untreated PD. JERK and time-domain measures showed the best test-retest reliability (JERK ICC was 0.86 in PD and 0.87 in controls; time-domain measures ICC ranged from 0.55 to 0.84 in PD and from 0.60 to 0.89 in controls). JERK, all but one time-domain measure, and one frequency measure were significantly correlated with the clinical postural stability score (r ranged from 0.50 to 0.63, 0.01 < *p* < 0.05). The JERK and time-domain measures showed the best reliability, while the frequency-domain measures had poorer test-retest reliability.

Regarding concurrent validity, most ACC measures of sway were significantly correlated with CoP measures of sway. Only two measures, mean velocity and mean frequency, were not significantly correlated with CoP. Several ACC measures showed significant correlation with the PIGD (postural instability and gait disability) sub-score of the UPDRS III. All except mean frequency, power and JERK were significantly and positively correlated with the PIGD sub-score related to clinical postural instability. No significant correlations were found between ACC measures and the total UPDRS-III.

The iTUG showed a significant difference in cadence between early PD and control subjects, as well as in angular velocity of arm-swing, turning duration and time to perform turn-to-sits [9].

##### Recommendation

To characterize posture control in PD, the most sensitive, reliable, and valid ISway measures that are recommend, are: 1) JERK, 2) RMS amplitude and mean velocity from the time-domain measures, and 3) centroidal frequency [8]. Among the subcomponents of iTUG, gait, turning and turn-to-sit were the most reliable and sit-to-stand was the least reliable [9].

#### Physilog®

##### Device description and outcomes that can be measured

Physilog is an ambulatory analysis method that uses body-attached gyroscopes to assess spatio-temporal parameters of gait, sway, physical activity, tremor and bradykinesia. Depending on the expected outcomes, one to seven inertial sensors, including accelerometer and gyroscopes, are used. Minimal attachment sites are used and no calibration is needed.

##### Clinimetric properties

Gait measurements using this device were performed in 10 people with PD with deep brain stimulation (DBS) [10]. PD patients had significantly different gait parameters compared to controls. Some of the gait parameters had high correlation with UPDRS subscores including stride length with a significant correlation (*r* = − 0.90) with UPDRS gait subscore. This algorithm was able to detect gait cycles and related gait events with very high sensitivity (>96 %) and with positive prediction value (PPV) > 98 %. The relative error in estimation of the gait cycle time was 2 % and for stride length and stride velocity < 8 %. These results have been demonstrated to be accurate enough to show significant differences between Stimulation ON and Stimulation OFF states in PD patients.

This system had a very high sensitivity in detecting gait cycles (100 % for controls and 100 % for PD patients during Stimulation ON and 99.6 % during Stimulation OFF). Sensitivity in detecting gait events was also very high (99.6 % for controls and 99.3 % for PD patients during Stimulation ON and 96.4 % during Stimulation OFF). The PPV in the detection of gait cycles was 100 % for controls, 98.9 % for Stimulation ON, and 98.4 for the Stimulation OFF group.

In another study [11], a novel symbol based symmetry index was calculated from this inertial sensor data. These measures were used to determine the symmetry of both upper and lower limbs during walking of 11 early-to-mid-stage PD patients and 15 controls. Sensitivity and specificity were high with an area under the curve (ROC) of 0.872 and also showed an excellent ICC of 0.949.

Additionally, algorithms to detect and quantify tremor, and quantify bradykinesia have been proposed and validated using the Physilog. One clinical study included 10 PD patients and 10 control subjects who undertook a 45 min protocol of 17 typical daily activities. The algorithm for tremor detection showed an overall sensitivity of 99.5 % and a specificity of 94.2 % in comparison to a video reference. The estimated tremor amplitude was highly correlated with the Unified Parkinson’s Disease Rating Scale (UPDRS) tremor subscore. There was a high and significant correlation between the estimated bradykinesia-related parameters estimated for the whole period of measurement and respective UPDRS subscores. Another clinical study assessed free movements of upper extremities of 11 PD patients for periods of 3 to 5 h. Correlations similar to those in the the first study were obtained. Moreover, one of the bradykinesia-related parameters showed significant correlation to UPDRS with window sizes as short as 5 min. This study showed that objective, accurate and simultaneous assessment of tremor and bradykinesia can be achieved in free movements of PD patients during their daily activities [12].

##### Recommendation

The system has been shown to successfully estimate gait parameters with a high degree of accuracy. The method has also been validated to assess gait changes in PD since the results obtained with these instrumental method reliably correlate with clinical scores obtained with commonly used scales, such as the UPDRS [10].

Regarding physical activity, kinematic features of the trunk movements were calculated during the transitions between sitting and standing postures. The proposed method showed a high sensitivity and specificity for the detection of basic body posture allocations: sitting, standing, lying, and walking periods, in PD patients and healthy individuals. Significant differences in parameters were found related to sit-to-stand and stand-to-sit transitions between PD patients and controls and also between PD patients with and without STN-DBS turned on. The method was shown to provide a simple, accurate, and effective means to objectively quantifying physical activities in both healthy subjects and PD patients, and possibility useful to assessing the level of motor functions in the latter [13].

#### StepWatch 3 (SAM)

##### Device description and outcomes that can be measured

The StepWatch 3 (SAM) is a step activity monitor used to capture differences in ambulatory activity according to age and functional limitation. The SAM is the size of a pager and attaches to the ankle. Once applied, it requires no maintenance by the user. The SAM is a microprocessor-linked unit that combines acceleration, position, and timing information to count complete gait cycles. The monitor records stride counts in 1-min intervals synchronized to a 24-h clock and stored on flash memory within the monitor. Stride counts are recorded as 0 during periods of inactivity. During activity the number of counts recorded per 1-min interval varies, depending primarily on the locomotor task characteristics.

Average daily values can be calculated for a number of steps, minutes of activity, number of activity bouts, variability of minute-to-minute activity, and randomness of minute-to-minute activity fluctuations.

##### Clinimetric properties

The validity, reliability and sensitivity of the SAM are supported by previous research in various diagnostic groups, and at different motor tasks including monitoring of walking and capture ambulatory activity decline in PD [14–17].

##### Recommendation

Step activity monitoring data has been shown to be useful for detecting differences in ambulatory activity according to age and functional limitation.

#### TriTrac RT3

##### Device description and outcomes that can be measured

The TriTrac RT3 accelerometer is a triaxial accelerometer that may be suitable for sustained tracking of free-living physical activity in the home environment. It is small, capable of collecting and storing data in one-minute epochs for 21 days, and has no external controls that can be manipulated during data collection.

##### Clinimetric properties

The accelerometer reliably measured physical activity (ICC, 0.85; 95 % confidence interval, 0.74–0.91). The standard error of measurement indicated that a second test would differ from a baseline test by ±23 %. Mean daily data collected in the first three days differed significantly from that of the mean daily data collected over seven days. The TriTrac RT3 appeared to distinguish level of mobility better than the seven-day recall questionnaire, and participants found the TriTrac RT3 to be a user-friendly and acceptable measure of physical activity [18].

##### Recommendation

The triaxial accelerometer was found to distinguish between people with PD with different levels of mobility. It was also well tolerated by participants.

#### McRoberts DynaPort

##### Device description and outcomes that can be measured

The DynaPort MiniMod Hybrid (The Hague, Netherlands; 87 × 45 × 14 mm, 74 g) combines acceleration sensors (range and resolution of ± 2 g and 0.001 g, respectively) and gyroscopes (range and resolution of ± 100 deg/s and 0.0069 deg/s, respectively), with six channels that assess the individual’s movement at 100Hz each. Using a widely available Bluetooth protocol, the DynaPort MiniMod Hybrid communicates wirelessly with a host Personal Computer.

Quantity measures included the total number of walking bouts, the percent of time spent walking which reflects the total walking in relation to the overall walking and non-walking activity, the total number of steps, median walking bout duration, median number of steps, and median cadence per bout. Quality-related sensor-derived measures included: frequency-derived measures that reflect variability of the gait pattern, regularity measures that reflect gait rhythmicity and consistency and the harmonic ratio, which is an index of gait smoothness. In addition, the study performed step-to-step analyses to evaluate the Phase Coordination Index, which is a measure of the consistency and accuracy of the left-right bilateral coordination during walking, i.e., timing of one foot with respect to the other [19].

##### Clinimetric properties

An algorithm was developed and successfully identified misstep events with a good hit ratio and specificity (odds ratio was 1.84, 95 % confidence intervals: 1.15–2.93) [20]. When applied to the three-day recording, misstep detections were associated with fall status and further supported the validity of the algorithm. The algorithms were able to detect even walking performed at relatively low speeds.

Weiss et. al. [19] demonstrated that PD fallers and non-fallers differ in their gait quality when evaluated in everyday settings, and Iluz et. al. [21] showed that PD fallers tend to produce about 23 % more missteps compare to the non-fallers.

##### Recommendation

The DynaPort MiniMod Hybrid can detect missteps during activities of daily living among patients with PD. The device is capable of measuring both acceleration and angular velocity in three planes and quantifying several mobility subtasks. It can be used clinically to monitor and promote physical activity [19].

The body-fixed sensors worn for three days can also be used to evaluate fall risk in patients with PD as they perform activities in their home and community settings.

#### Axivity (AX3)

##### Device description and outcomes that can be measured

The Axivity (AX3) is a three-axis accelerometer that has a non-volatile flash memory chip linked by a USB-enabled microcontroller. Inside the sealed polycarbonate puck is a temperature sensor, ambient light sensor, and real time clock and lithium polymer battery. The sensor can record up to 21 days of continuous data. The device is suitable for use in a variety of environments and is water resistant (up to 1.5 m).

Comparing data collected over seven days with data collected in the laboratory during scripted tests showed that data collected from gait in free-living conditions can discriminate disease better than laboratory- based data.

##### Clinimetric properties

Axivity (AX3) was used in a study of 30 people with PD and 30 healthy age-matched controls; 14 gait characteristics were quantified. Of the 14 gait characteristics compared, agreement between instruments was excellent for 4 (ICCs 0.913–0.983); moderate for 4 (ICCs 0.508–0.766); and poor for 6 characteristics (ICCs −0.637–0.370). Further analysis revealed that differences reflect an increased sensitivity of accelerometry in detecting motion, rather than measurement error. The increased sensitivity shown for these characteristics may be of particular interest to researchers wanting to obtain and interpret free world gait data [22].

In a pilot study, 14 body-worn monitor-based outcomes were presented across the gait subdomains, which include magnitude, frequency and spatio-temporal characteristics. Body-worn monitor outcomes were compared with manually recorded values, no significant differences were reported between locomotion and TUG tasks. Significant differences were found for the total distance walked during endurance and times for repeated sit-to-stand-to-sit transitions. These findings supported the feasibility of this method in enhancing measurement of physical capacity, making its application useful in a range of intervention-based studies or pathological assessments [23].

Importantly, it was shown that fallers tended to walk in shorter bouts and had a less variable walking pattern compared to non-fallers. People with PD spent less time walking, took fewer steps, and accumulated proportionally more steps in shorter bouts compared to the elderly, regardless of falls history. Preliminary results showed that there is an association between falls history and physical activity [22].

##### Recommendation

Use of a body-worn monitor is recommended for the measurement of gait. It is likely to produce more sensitive data for asymmetry and variability features. These results support the use of a single accelerometer-based sensor to assess falls risk in free living settings and to potentially predict falls.

### Non-wearable devices

#### Ground platforms

##### Wii Balance Board

Device description and outcomes that can be measured

Nintendo Wii Balance Board (WBB) is a commonly used accessory for the Wii video game consoles. WBB is considered a cheap, widespread, clinimetric robust device that can be used to measure postural instability in PD patients. It consists of a ground platform, which has four pressure sensors that analyze force distribution and measure CoP movement. It has a small usable area, which can limit to some extent postural assessment in individuals with wider upright stance, and the sensors’ accuracy weight is limited to 150 kg.

The use of WBB has been proposed as an assessment tool for postural instability in PD patients. Additionally, some rehabilitation programs using the WBB addressing balance impairment have been studied with promising results [24].

Clinimetric properties

Test-retest reliability was high (ICCs > 0.75) for CoP path length in 3/4 balance tasks (single limb, eyes open: ICC = 0.86; single limb, eyes closed: ICC = 0.81; double limb, eyes open: ICC = 0.66 and double limb, eyes closed: ICC = 0.91), [25].

Concurrent validity (between WBB and AMTI Model) was excellent in balance tasks and also among test sessions with ICC ranging from 0.77 to 0.89 [25]. In a more recent study [26] with 20 PD patients, the values of ICC were higher (ICC = 0.92-0.98) due to signal processing improvement.

Sensitivity to change of the WBB was considered high, with standard error measurement (SEM) between 8.7–13.1 % and minimum detectable change (MDC) ranging from 24.5–29.4 %. However, WBB yielded higher values for SEM and MDC than a laboratory force plate in 3 out of 4 trials (8.7–13.1 % for the WBB versus 5.3–13.2 % for the Force Plate concerning the SEM values, and 24.5–29.4 % for the WBB versus 14.5–34.7 % for the Force Plate in regard to the MDC values) [25].

Recommendation

WBB is considered a cheap, widespread, clinimetric robust device that can be used to measure postural instability in patients with PD. WBB is recommend for home monitoring of PD patients’ signs. However, some limitations can be identified such as the small usable area, which can limit postural assessment in individuals with wider upright stance, and the sensors’ accuracy weight limit.

##### GAITRite®

Device description and outcomes that can be measured

The GAITRite® system is an electronic pathway that contains pressure sensitive sensors arranged in a grid-like pattern. There are several versions of the GAITRite® device available with different lengths. The carpet is portable and can be rolled up for transportation. It is used for laboratory evaluation and provides information regarding several gait parameters, such as walking speed, cadence and step length. It has been used to assess the degree of axial bradykinesia and the effect of L-dopa therapy on gait variables in PD [27]. Some safety precautions have been highlighted as needed, such as a wall fixed side rail or supervised gait.

Clinimetric properties

Test-retest reliability on 31 older healthy people was assessed in two sessions with a two-week interval. The ICCs were high for walking speed, cadence, step length and left toe in/out angle (between 0.82 and 0.91), and moderate for base of support (0.49 for the left and 0.56 for the right) [28]. Coefficient of variation was small for speed, cadence and step length (3.1–3.5 %), but relatively large for base of support (14.3–15.2 %) and toe in/out angles (24.4–33.0 %) [28].

The relation between GAITRite®-derived gait variables and UPDRS scores and timed tests regarding therapeutic efficiency was examined in 13 patients with PD [27]. Significant correlations were found between L-dopa improvement in gait parameters and the UPDRS III score in the following variables: velocity, stride length, swing phase ratio, and stance phase ratio and double support ratio [27].

Using GAITRite® and self-report questionnaires in a population of 241 elderly healthy individuals, preliminary recommended parameters for clinical meaningful change are 0.001 s for stance time and swing time and 0.25 cm for step length SD [29].

Importantly, we highlight that these clinimetric proprieties refer to the standard GAITRite® walkway and should not be extrapolated to other lengths of this instrument.

Recommendation

The GAITRite® system is a reliable instrument, with extended validation for bradykinesia and the therapeutic effect of L-dopa. Further analysis of sensitivity to change parameters is needed to strengthen its clinimetric properties. Nonetheless, GAITRite® fulfills the proposed criteria and is considered “recommended” for monitoring PD motor signs.

### Hybrid technologies

#### Network platforms and telemedicine

##### Kinesia™

Device description and outcomes that can be measured

Kinesia™ integrates accelerometers and gyroscopes in a compact patient-worn unit to capture kinematic movement disorder features. The sensor component of the device is mounted on a ring, which fits on a finger. The sensor component conveys signal data by wire to a wrist-mounted component, which wirelessly transmits data to a local Computer. The Kinesia™ device has a range limited to 30 m for wireless coverage.

The Kinesia™ system successfully demonstrated the capacity to ascertain tremor with increased time resolution, which could aid in evaluating the efficacy of treatment protocols and improve patient management. Importantly, 45 % of the subject group indicated they would prefer not to wear the device in public.

Clinimetric properties

In one study [30], individuals with PD performed the tremor subset of the UPDRS III while wearing Kinesia™. Quantitative kinematic features were processed and highly correlated to clinician scores for rest tremor (*r*^(2)^ = 0.89), postural tremor (*r*^(2)^ = 0.90), and kinetic tremor (*r*^(2)^ = 0.69). The quantitative features were used to develop a mathematical model that predicted tremor severity scores for new data with low errors [30].

Recommendation

The Kinesia™ sensor component is a small, wrist-mounted component that is able to convey data wirelessly to a local PC with up to 30 m of wireless coverage. The device has been shown to be able to successfully ascertain tremor. However, it suffered from poor subject acceptability.

## Discussion and conclusions

From 168 articles included in this study, research has yielded 22 wearable devices, 38 non-wearable devices, and 13 instruments with combined technology used for monitoring PD clinical manifestations. Despite the common general outcomes, these devices encompass diverse variables and their availability and clinical suitability differ.

Of these 73 devices, 30 were classified as “listed” due to the lack of external usage or satisfactory clinimetric properties. Some are self-developed, original devices and their availability for use by other developers is thus reduced. Despite using commercially available technology, in some cases, the high cost limits access to certain devices. Furthermore, some of the experimental conditions are difficult to replicate and occasionally the product was not commercially available. Within the “listed” category there are instruments which have undergone different degrees of clinimetric testing and further testing may be considered.

Thirty-four out of 73 instruments assessed were ranked as “suggested”. These devices are based on commercially available technology used either in scientific laboratories (inertial measurement units, force plates, infrared cameras, radio waves) or by the common citizen (videogame consoles, tablets, microphones), which can explain their broader use.

A complete clinimetric evaluation was not available or any of the “suggested” devices: incomplete clinimetric analysis was mostly due to lack of information related to sensitivity over time, followed by an inexistent or unsuitable reliability analysis. As in the listed category, the extent of the clinimetric evaluation varies for each device and further research may provide the missing parameters enabling promotion.

Finally, nine devices were labeled as “recommended” as they fulfill all the criteria. To facilitate follow-up on the recommend devices we highlight that the contact of the suppliers of each device can be easily obtained through basic Google search.

Importantly, one of the limitations of this review is our inability to comment on the *Minimal Clinically Difference* (MCD) of each device given the lack of information regarding this clinimetric property. Additionally, grouping all types of validity in to a single yes/no binary answer may not accurately reflect the maturity/validity of a certain system given the different types of validity and many degrees of validity that exist. It should, however, be a very relevant issue to consider for future studies. In fact, the validity of a given system can be a complex issue and deserves further reflection. In many studies systems measuring different signals are compared when doing the validation such as acceleration versus pressure versus position. When assessing the systems, researchers should be alerted to the nature of the comparison for the validation.

Importantly, for a number of the devices, the same signals, with similar levels of “quality”, are available. For example, 3D accelerations of the lower back can be measured accurately and reliably with McRoberts, Axivity and APDM. Metrics that depend on this signal can likely be obtained with any of these devices, even if a validation study was performed only on one device and not the other. Thus, many of the recommendations can be applied more broadly. Similarly, questions about MCD are generally not device dependent, but signal/metric dependent.

In conclusion, objective sensing technology is of growing interest in the study of PD, yet, one of the remaining controversies in the use of these devices is their clinimetric properties and testing. Considerable attention has been growing with regard to the reliability and validity testing of new scales, and sensing technology should not be an exception to this. We believe that until existing devices are better tested clinimetrically, there is little to gain in the further development of new devices. At this moment, we can briefly summarize that the PD symptoms that can be objectively measured using the reviewed devices are postural control, tremor, bradykinesia, freezing, dyskinesia, gait, and daily activity/physical activity. Future studies should focus more on disease progression markers and non-motor symptoms such as cognition, sleep and dysphagia.

## Additional file

Additional file 1: Table S1. Overview of the instruments assessed and their classification. (DOCX 238 kb)

## Abbreviations

ACC: acceleration
ADL: activity daily live
APDM: Ambulatory Parkinson’s Disease Monitoring
CDMS: clinical data management system
CoP: center of pressure
CTR: control
FOG: freezing of gait
ICC: intra class coefficient
iTUG: instrumented timed up and go
MDC: minimum detectable change
PD: Parkinson’s disease
RoM: range of motion
SEM: standard error measurement.
UPDRS: Unified Parkinson Disease Rating Scale
