# Erratum to: A systematic review of the characteristics and validity of monitoring technologies to assess Parkinson’s disease

**DOI:** 10.1186/s12984-016-0181-2

**Published:** 2016-08-01

**Authors:** Catarina Godinho, Josefa Domingos, Guilherme Cunha, Ana T. Santos, Ricardo M. Fernandes, Daisy Abreu, Nilza Gonçalves, Helen Matthews, Tom Isaacs, Joy Duffen, Ahmed Al-Jawad, Frank Larsen, Artur Serrano, Peter Weber, Andrea Thoms, Stefan Sollinger, Holm Graessner, Walter Maetzler, Joaquim J. Ferreira

**Affiliations:** 1 Instituto de Medicina Molecular, Faculdade de Medicina, Universidade de Lisboa, Avenida Professor Egas Moniz, Lisboa, 1649-028 Portugal; 2 Laboratory of Clinical Pharmacology and Therapeutics, Faculty of Medicine, University of Lisbon, Lisbon, Portugal; 3 Instituto Superior de Ciências da Saúde Egas Moniz, Center for Interdisciplinary Research Egas Moniz (CiiEM), Monte de Caparica, Portugal; 4 CNS-Campus Neurológico Sénior, Torres Vedras, Portugal; 5 The Cure Parkinson’s Trust, London, UK; 6 HSG-IMIT, Villingen-Schwenningen, Germany; 7 Norwegian Centre for Telemedicine, Tromso, Norway; 8 Hasomed GmbH, Magdeburg, Germany; 9 AbilityNet, London, UK; 10 Institute for Medical Genetics and Applied Genomics, University of Tuebingen, Tuebingen, Germany; 11 Department of Neurodegeneration, Hertie Institute for Clinical Brain Research, Center of Neurology, University of Tuebingen, Tuebingen, Germany

## Erratum

Unfortunately, the PDF and the XML of the original version of this article [1] contained an error and the references in the reference list starting from the second reference [21] were wrongly numbered:

- the second reference [21], Del Din et al. (2015), should be reference [22];
- reference [22], Godfrey et al. (2015), should be [23];
- reference [23], Esculier et al. (2012), should be [24];
- reference [24], Clark et al. (2010), should be [25];
- reference [25], Holmes et al. (2013), should be [26];
- reference [26], Chien et al. (2006), should be [27];
- reference [27], Menz et al. (2004), should be [28];
- reference [28], Brach et al. (2010), should be [29];
- and reference [29], Giuffrida et al. (2009), should be [30].

Please refer to the HTML of the original article [1] for the correct reference list: http://jneuroengrehab.biomedcentral.com/articles/10.1186/s12984-016-0136-7.

## References

[1] Godinho C, Domingos J, Cunha G, Santos AT, Fernandes RM, Abreu D et al. A systematic review of the characteristics and validity of monitoring technologies to assess Parkinson’s disease. J Neuroeng Rehabil. 2015;13(24). doi:10.1186/s12984-016-0136-7 10.1186/s12984-016-0136-7 PMC4788909 26969628

