# Supplementary material for: A systematic review of the characteristics and validity of monitoring technologies to assess Parkinson’s disease
Source: J Neuroeng Rehabil. 2016 Mar 12;13:24. doi: 10.1186/s12984-016-0136-7 (PMC4788909; doi:10.1186/s12984-016-0136-7)
Supplement: Additional file 1: Table S1. — Overview of the instruments assessed and their classification. (DOCX 238 kb) [file 12984_2016_136_MOESM1_ESM.docx]

Supplemental table - Overview of the instruments assessed and their classification

| Device | Clinical  Parameter | External usage | Clinimetric properties | | | Classification |
| --- | --- | --- | --- | --- | --- | --- |
|  |  |  | **Reliability** | **Validity** | **Sensitivity to change** |  |
| Wearable Devices | | | | | | |
| Mobility Lab System  (APDM)  [[1-3](#_ENREF_1)] [[4](#_ENREF_4)] | Bradykinesia  Dyskinesia  Postural control  Gait/FOG  ADLs | **X** | **X** | **X** | **X** | Recommended |
| Physilog®  [[5-7](#_ENREF_5)] [[8](#_ENREF_8)] [[9](#_ENREF_9)] | Postural control  Gait/FOG  Physical Activity  Tremor  Bradykinesia | **X** | **X** | **X** | **X** | Recommended |
| StepWatch 3  (SAM)  [[10-14](#_ENREF_10)] | ADLs  Gait | **X** | **X** | **X** | **X** | Recommended |
| TriTrac RT3  [[15](#_ENREF_15)] | ADLs | **X** | **X** | **X** | **X** | Recommended |
| McRoberts Dynaport  [[16](#_ENREF_16)] [[17](#_ENREF_17)] [[18](#_ENREF_18)] [[19](#_ENREF_19)] [[20](#_ENREF_20)] | ADLs  Falls/ near Falls  Gait | **X** | **X** | **X** | **X** | Recommended |
| Axivity (AX3)  [[21](#_ENREF_21)] [[22](#_ENREF_22)] [[23](#_ENREF_23)] [[24](#_ENREF_24)] | Physical Activity/ADLs  Falls  Gait | **X** | **X** | **X** | **X** | Recommended |
| Xsens MTx and Philips Pi-Node  [[25](#_ENREF_25), [26](#_ENREF_26)] | Gait/FOG | **X** | **X** | **X** | **−** | Suggested |
| Lifecorder^®^  (Suzuken Co.)  [[27](#_ENREF_27)] | Gait/FOG  ADLs | **X** | **X** | **−** | **−** | Suggested |
| iPhone  [[28](#_ENREF_28)] | Tremor  ADLs | **X** | **X** | **−** | **X** | Suggested |
| Vitaport  Activity Monitor^®^  [[29](#_ENREF_29), [30](#_ENREF_30)] | Tremor  Bradykinesia  Dyskinesia  Gait/FOG  ADLs | **X** | **−** | **X** | **X** | Suggested |
| Kionix (KXPA4-2050) [[31](#_ENREF_31)] | Gait | **X** | **X** | **−** | **X** | Suggested |
| ActiTrac®  [[32](#_ENREF_32)] | Bradykinesia  Dyskinesia | **X** | **X** | **−** | **−** | Suggested |
| Actiwatch  (AW-64)  [[33-35](#_ENREF_33)] | ADLs | **X** | **−** | **X** | **−** | Suggested |
| ANCO  [[36-38](#_ENREF_36)] | Dyskinesia  FOG | **X** | **−** | **X** | **−** | Suggested |
| Finger tapping system [[39-41](#_ENREF_39)] | Bradykinesia | **X** | **−** | **X** | **−** | Suggested |
| ActivPAL  [[42](#_ENREF_42)] | Sedentary  Behavior | **X** | **X** | **_** | **_** | Suggested |
| Smart watch  (WIMM One)  [[43](#_ENREF_43)] | Tremor |  | **X** |  | **X** | Listed |
| Inertial Measurement Unit  [[44](#_ENREF_44), [45](#_ENREF_45)] | Gait  FOG | **−** | **X** | **−** | **X** | Listed |
| Virtual reality data glove  [[46](#_ENREF_46)] | Grip Force | **−** | **−** | **−** | **−** | Listed |
| Portable Gait Rhythmogram  [[47](#_ENREF_47)] | Bradykinesia  Postural control  Gait/FOG | **−** | **−** | **−** | **X** | Listed |
| W-MAS  [[48](#_ENREF_48)] | Gait | **−** | **X** | **−** | **−** | Listed |
| Tremorometer  [[49](#_ENREF_49)] | Tremor | **−** | **X** | **X** | **−** | Listed |
| Non-Wearable Devices | | | | | | |
| Wii Balance Board  [[50-52](#_ENREF_50)] | Postural instability | **X** | **X** | **X** | **X** | Recommended |
| GAITRite  [[53-55](#_ENREF_53)] | Bradykinesia (axial)  Gait/FOG | **X** | **X** | **X** | **X** | Recommended |
| AMTI  [[56-59](#_ENREF_56)] | Postural instability  Gait/FOG | **X** | **X** | **X** | **−** | Suggested |
| Balance Master  [[60-63](#_ENREF_60)] | Postural instability | **X** | **X** | **X** | **−** | Suggested |
| Inclinometric pendulum  [[64-66](#_ENREF_64)] | Postural instability | **X** | **X** | **X** | **−** | Suggested |
| QFP Medicapteurs  [[67](#_ENREF_67), [68](#_ENREF_68)] | Postural instability | **X** | **−** | **X** | **−** | Suggested |
| Stabilometer  [[69-71](#_ENREF_69)] | Postural instability | **X** | **−** | **−** | **−** | Suggested |
| Digital Tablets | | | | | | |
| Intuos Wacom  [[72](#_ENREF_72), [73](#_ENREF_73)] | Tremor  Bradykinesia | **X** | **X** | **X** | **−** | Suggested |
| Easypen C6  [[74](#_ENREF_74), [75](#_ENREF_75)] | Tremor  Dyskinesia | **X** | **−** | **X** | **−** | Suggested |
| TDS Zed pen  [[76](#_ENREF_76)] | Handwriting | **−** | **−** | **−** | **−** | Listed |
| Piano keyboards | | | | | | |
| Quantitative Digitography  [[77-81](#_ENREF_77)] | Tremor  Freezing | **−** | **X** | **X** | **−** | Listed |
| Video Recording cameras | | | | | | |
| Vicon  [[82-85](#_ENREF_82)] | Tremor  Gait/FOG | **X** | **X** | **X** | **−** | Suggested |
| Elite System  [[86-88](#_ENREF_86)] | Gait  Posture | **X** | **−** | **−** | **−** | Suggested |
| Non-rigid image registration camera  [[89](#_ENREF_89), [90](#_ENREF_90)] | Dyskinesia | **−** | **−** | **X** | **−** | Listed |
| Camera Smart BTS  [[91](#_ENREF_91)] | Gait | **−** | **−** | **−** | **−** | Listed |
| Computerized video by Tsinghua University  [[92](#_ENREF_92)] | Tremor  Rigidity | **−** | **−** | **−** | **−** | Listed |
| Infrared camera Optotrak 3020  [[93](#_ENREF_93)] | Gait | **−** | **−** | **−** | **−** | Listed |
| Passive marker-based analyser of movement  [[94](#_ENREF_94)] | Finger tapping | **−** | **−** | **−** | **−** | Listed |
| Ultrasound | | | | | | |
| Movement analysis CMS 50; Zebris  [[95-98](#_ENREF_95)] | Hand movements | **X** | **X** | **X** | **−** | Suggested |
| Electronic Appliances (of various origins) connected to a computational unit | | | | | | |
| Wii Remote  [[99-102](#_ENREF_99)] | Tremor | **X** | **−** | **−** | **−** | Suggested |
| Hand held transducer  [[103](#_ENREF_103)] | Bradykinesia | **−** | **−** | **X** | **−** | Listed |
| Joystick/steering wheel Saitek  [[104](#_ENREF_104)] | Bradykinesia | **−** | **−** | **X** | **−** | Listed |
| Spiralometry  [[105](#_ENREF_105)] | Tremor | **−** | **−** | **X** | **−** | Listed |
| Computer software | | | | | | |
| BRAIN test  [[106](#_ENREF_106), [107](#_ENREF_107)] | Bradykinesia | **X** | **−** | **X** | **−** | Suggested |
| Web version of BEP I [[108](#_ENREF_108)] | Bradykinesia | **−** | **−** | **X** | **−** | Listed |
| Computer-based assessment tool  [[109](#_ENREF_109), [110](#_ENREF_110)] | Tremor  Bradykinesia | **−** | **−** | **−** | **−** | Listed |
| Microphones | | | | | | |
| Microphones by Plantronics  [[111](#_ENREF_111), [112](#_ENREF_112)] | Voice/Speech | **X** | **−** | **X** | **−** | Suggested |
| Microphones by AKG  [[113](#_ENREF_113), [114](#_ENREF_114)] | Speech | **X** | **−** | **X** | **−** | Suggested |
| Shure model SM 58 [[115](#_ENREF_115)] | Speech | **−** | **−** | **−** | **−** | Listed |
| Microphone Tandy [[116](#_ENREF_116)] | Saliva/Swallowing | **−** | **−** | **−** | **−** | Listed |
| Dynamic microphone  [[117](#_ENREF_117)] | Voice | **−** | **−** | **−** | **−** | Listed |
| Dynamometers and force transducers | | | | | | |
| CYBEX II Dynamometer  [[118-121](#_ENREF_118)] | Muscle Strength  Articular Speed/Range of motion | **X** | **X** | **−** | **−** | Suggested |
| Force transducers [[122](#_ENREF_122), [123](#_ENREF_123)] | Lip closure force | **X** | **X** | **−** | **−** | Suggested |
| Mechanical test ring [[124](#_ENREF_124)] | Upper limb stiffness | **−** | **−** | **X** | **−** | Listed |
| The hardware-program complex [[125](#_ENREF_125)] | Eyes, head and hand movements | **−** | **−** | **−** | **−** | Listed |
| Magnetic induction | | | | | | |
| Electromagnetic articulography  [[126](#_ENREF_126), [127](#_ENREF_127)] | Lingual movements | **X** | **−** | **−** | **−** | Suggested |
| Magnetic sensing system  [[128-130](#_ENREF_128)] | Finger movements | **−** | **X** | **X** | **−** | Listed |
| Radar | | | | | | |
| Continuous wave radar  [[131](#_ENREF_131)] | Gait | **−** | **−** | **−** | **−** | Listed |
| Hybrid technologies  Network Platforms and Telemedicine | | | | | | |
| Kinesia™  [[132](#_ENREF_132)] | Tremor | **X** | **X** | **X** | **X** | Recommended |
| Mercury Network  Platform  (SHIMMER sensors) [[133-137](#_ENREF_133)] [[138](#_ENREF_138)] | Tremor  Bradykinesia  Dyskinesia | **X** | **X** | **−** | **X** | Suggested |
| PERFORM  [[139-141](#_ENREF_139)] [[142](#_ENREF_142)] [[143](#_ENREF_143)] | Tremor  Bradykinesia  Dyskinesia  FOG  Gait | **X** | **X** | **X** | **_** | Suggested |
| Intel AHTD home system  [[144](#_ENREF_144), [145](#_ENREF_145)] | Tremor  Dysphonia | **X** | **X** | **−** | **−** | Suggested |
| Telemedicine Instrument  (ACC (3- axis ADXL330) + PDA + Internet connection + Hospital Unit)  [[146](#_ENREF_146)] | Tremor | **−** | **X** | **−** | **X** | Listed |
| Network of Sensor Nodes  (iNODEs + FSR + Wireless communication + Plethysmography) [[147](#_ENREF_147)] | Locomotor-respiration coordination | **−** | **−** | **−** | **−** | Listed |
| Others | | | | | | |
| ACC (MeacX) + sEMG (M-00-S)  [[148-150](#_ENREF_148)] | Tremor  FOG | **X** | **−** | **X** | **−** | Suggested |
| Trigno System  [[151](#_ENREF_151)] | Tremor  Bradykinesia | **X** | **−** | **X** | **−** | Suggested |
| Diadochokinesimeter [[152](#_ENREF_152), [153](#_ENREF_153)] | Bradykinesia | **X** | **X** | **−** | **−** | Suggested |
| Mini-motionlogger Actigraph + 24-hour ECG recording  [[154](#_ENREF_154)] [[155](#_ENREF_155)] [[156](#_ENREF_156)] | Sleep  Physical Activity | **X** | **−** | **X** | **−** | Suggested |
| Wearable assistant [[157](#_ENREF_157)] | FOG | **−** | **−** | **X** | **−** | Listed |
| Wireless EMG  [[158](#_ENREF_158)] | Tremor  Bradykinesia  Rigidity | **−** | **−** | **−** | **−** | Listed |
| Force Sensitive Resistors (FSR) + ACC (3-axis ADXL330) + Video  [[159](#_ENREF_159)] | FOG | **−** | **X** | **−** | **X** | Listed |

(X) Information available, (**−**) no information available.

1. Mancini M, Horak FB, Zampieri C, Carlson-Kuhta P, Nutt JG, Chiari L. Trunk accelerometry reveals postural instability in untreated Parkinson's disease. Parkinsonism Relat Disord. 2011;17(7):557-62. doi:S1353-8020(11)00142-8

2. Mancini M, Salarian A, Carlson-Kuhta P, Zampieri C, King L, Chiari L et al. ISway: a sensitive, valid and reliable measure of postural control. J Neuroeng Rehabil. 2012;9:59. doi:1743-0003-9-59

3. Mancini M, Carlson-Kuhta P, Zampieri C, Nutt JG, Chiari L, Horak FB. Postural sway as a marker of progression in Parkinson's disease: A pilot longitudinal study. Gait Posture. 2012. doi:S0966-6362(12)00138-5

4. Salarian A, Horak FB, Zampieri C, Carlson-Kuhta P, Nutt JG, Aminian K. iTUG, a sensitive and reliable measure of mobility. IEEE Trans Neural Syst Rehabil Eng. 2010;18(3):303-10. doi:10.1109/TNSRE.2010.2047606.

5. Salarian A, Russmann H, Vingerhoets FJ, Dehollain C, Blanc Y, Burkhard PR et al. Gait assessment in Parkinson's disease: toward an ambulatory system for long-term monitoring. IEEE Trans Biomed Eng. 2004;51(8):1434-43. doi:10.1109/TBME.2004.827933.

6. Salarian A, Zampieri C, Horak FB, Carlson-Kuhta P, Nutt JG, Aminian K. Analyzing 180 degrees turns using an inertial system reveals early signs of progression of Parkinson's disease. Conf Proc IEEE Eng Med Biol Soc. 2009;2009:224-7. doi:10.1109/IEMBS.2009.5333970.

7. Sant'Anna A, Salarian A, Wickstrom N. A new measure of movement symmetry in early Parkinson's disease patients using symbolic processing of inertial sensor data. IEEE Trans Biomed Eng. 2011;58(7):2127-35. doi:10.1109/TBME.2011.2149521.

8. Salarian A, Russmann H, Wider C, Burkhard PR, Vingerhoets FJ, Aminian K. Quantification of tremor and bradykinesia in Parkinson's disease using a novel ambulatory monitoring system. IEEE Trans Biomed Eng. 2007;54(2):313-22. doi:10.1109/TBME.2006.886670.

9. Salarian A, Russmann H, Vingerhoets FJ, Burkhard PR, Aminian K. Ambulatory monitoring of physical activities in patients with Parkinson's disease. IEEE Trans Biomed Eng. 2007;54(12):2296-9.

10. Shepherd EF, Toloza E, McClung CD, Schmalzried TP. Step activity monitor: increased accuracy in quantifying ambulatory activity. J Orthop Res. 1999;17(5):703-8. doi:10.1002/jor.1100170512.

11. Munneke M, de Jong Z, Zwinderman AH, Tijhuis GJ, Hazes JM, Vliet Vlieland TP. The value of a continuous ambulatory activity monitor to quantify the amount and intensity of daily activity in patients with rheumatoid arthritis. J Rheumatol. 2001;28(4):745-50.

12. Cavanaugh JT, Coleman KL, Gaines JM, Laing L, Morey MC. Using step activity monitoring to characterize ambulatory activity in community-dwelling older adults. J Am Geriatr Soc. 2007;55(1):120-4.

13. Speelman AD, van Nimwegen M, Borm GF, Bloem BR, Munneke M. Monitoring of walking in Parkinson's disease: validation of an ambulatory activity monitor. Parkinsonism Relat Disord. 2011;17(5):402-4. doi:S1353-8020(11)00048-4

14. Cavanaugh JT, Ellis TD, Earhart GM, Ford MP, Foreman KB, Dibble LE. Capturing ambulatory activity decline in Parkinson's disease. J Neurol Phys Ther. 2012;36(2):51-7.

15. Hale LA, Pal J, Becker I. Measuring free-living physical activity in adults with and without neurologic dysfunction with a triaxial accelerometer. Arch Phys Med Rehabil. 2008;89(9):1765-71. doi:S0003-9993(08)00429-2

16. Palmerini L, Rocchi L, Mellone S, Valzania F, Chiari L. Feature selection for accelerometer-based posture analysis in Parkinson's disease. IEEE Trans Inf Technol Biomed. 2011;15(3):481-90. doi:10.1109/TITB.2011.2107916.

17. Iluz T, Gazit E, Herman T, Sprecher E, Brozgol M, Giladi N et al. Automated detection of missteps during community ambulation in patients with Parkinson's disease: a new approach for quantifying fall risk in the community setting. J Neuroeng Rehabil. 2014;11:48. doi:10.1186/1743-0003-11-48.

18. Weiss A, Brozgol M, Dorfman M, Herman T, Shema S, Giladi N et al. Does the evaluation of gait quality during daily life provide insight into fall risk? A novel approach using 3-day accelerometer recordings. Neurorehabil Neural Repair. 2013;27(8):742-52. doi:10.1177/1545968313491004.

19. Herman T, Weiss A, Brozgol M, Giladi N, Hausdorff JM. Gait and balance in Parkinson's disease subtypes: objective measures and classification considerations. J Neurol. 2014;261(12):2401-10. doi:10.1007/s00415-014-7513-6.

20. Weiss A, Herman T, Giladi N, Hausdorff JM. Objective assessment of fall risk in Parkinson's disease using a body-fixed sensor worn for 3 days. PLoS One. 2014;9(5):e96675. doi:10.1371/journal.pone.0096675.

21. Godfrey A, Del Din S, Barry G, Mathers JC, Rochester L. Instrumenting gait with an accelerometer: a system and algorithm examination. Med Eng Phys. 2015;37(4):400-7. doi:10.1016/j.medengphy.2015.02.003.

22. Del Din S, Godfrey A, Coleman S, Galna B, Lord S, Rochester L. Time-dependent changes in postural control in early Parkinson's disease: what are we missing? Med Biol Eng Comput. 2015. doi:10.1007/s11517-015-1324-5.

23. Del Din S, Godfrey A, Rochester L. Validation of an accelerometer to quantify a comprehensive battery of gait characteristics in healthy older adults and Parkinson's disease: toward clinical and at home use. IEEE journal of biomedical and health informatics. 2015. doi:10.1109/JBHI.2015.2419317.

24. Godfrey A, Lara J, Munro CA, Wiuff C, Chowdhury SA, Del Din S et al. Instrumented assessment of test battery for physical capability using an accelerometer: a feasibility study. Physiol Meas. 2015;36(5):N71-83. doi:10.1088/0967-3334/36/5/N71.

25. Gonzalez RC, Lopez AM, Rodriguez-Uria J, Alvarez D, Alvarez JC. Real-time gait event detection for normal subjects from lower trunk accelerations. Gait Posture. 2010;31(3):322-5. doi:S0966-6362(09)00666-3

26. Esser P, Dawes H, Collett J, Feltham MG, Howells K. Validity and inter-rater reliability of inertial gait measurements in Parkinson's disease: a pilot study. J Neurosci Methods. 2012;205(1):177-81. doi:S0165-0270(12)00007-6

27. Saito N, Yamamoto T, Sugiura Y, Shimizu S, Shimizu M. Lifecorder: a new device for the long-term monitoring of motor activities for Parkinson's disease. Intern Med. 2004;43(8):685-92.

28. Lemoyne R, Mastroianni T, Cozza M, Coroian C, Grundfest W. Implementation of an iPhone for characterizing Parkinson's disease tremor through a wireless accelerometer application. Conf Proc IEEE Eng Med Biol Soc. 2010;2010:4954-8. doi:10.1109/IEMBS.2010.5627240.

29. Keijsers NL, Horstink MW, van Hilten JJ, Hoff JI, Gielen CC. Detection and assessment of the severity of levodopa-induced dyskinesia in patients with Parkinson's disease by neural networks. Mov Disord. 2000;15(6):1104-11.

30. Lord S, Rochester L, Baker K, Nieuwboer A. Concurrent validity of accelerometry to measure gait in Parkinsons Disease. Gait Posture. 2008;27(2):357-9.

31. Yang CC, Hsu YL, Shih KS, Lu JM. Real-Time Gait Cycle Parameter Recognition Using a Wearable Accelerometry System. Sensors (Basel). 2011;11(8):7314-26.

32. Garcia Ruiz PJ, Sanchez Bernardos V. Evaluation of ActiTrac (ambulatory activity monitor) in Parkinson's Disease. J Neurol Sci. 2008;270(1-2):67-9. doi:S0022-510X(08)00084-1

33. Stavitsky K, Saurman JL, McNamara P, Cronin-Golomb A. Sleep in Parkinson's disease: a comparison of actigraphy and subjective measures. Parkinsonism Relat Disord. 2010;16(4):280-3.

34. Stavitsky K, Cronin-Golomb A. Sleep quality in Parkinson disease: an examination of clinical variables. Cogn Behav Neurol. 2011;24(2):43-9. doi:10.1097/WNN.0b013e31821a4a95.

35. Stavitsky K, Neargarder S, Bogdanova Y, McNamara P, Cronin-Golomb A. The impact of sleep quality on cognitive functioning in Parkinson's disease. J Int Neuropsychol Soc. 2012;18(1):108-17.

36. Tsipouras MG, Tzallas AT, Rigas G, Bougia P, Fotiadis DI, Konitsiotis S. Automated Levodopa-induced dyskinesia assessment. Conference proceedings : Annual International Conference of the IEEE Engineering in Medicine and Biology Society IEEE Engineering in Medicine and Biology Society Conference. 2010;2010:2411-4. doi:10.1109/IEMBS.2010.5626130.

37. Tripoliti EE, Tzallas AT, Tsipouras MG, Rigas G, Bougia P, Leontiou M et al. Automatic detection of freezing of gait events in patients with Parkinson's disease. Computer methods and programs in biomedicine. 2013;110:12-26. doi:10.1016/j.cmpb.2012.10.016.

38. Tsipouras MG, Tzallas AT, Rigas G, Tsouli S, Fotiadis DI, Konitsiotis S. An automated methodology for levodopa-induced dyskinesia: assessment based on gyroscope and accelerometer signals. Artificial intelligence in medicine. 2012;55:127-35. doi:10.1016/j.artmed.2012.03.003.

39. Okuno R, Yokoe M, Fukawa K, Sakoda S, Akazawa K. Measurement system of finger-tapping contact force for quantitative diagnosis of Parkinson's disease. Conference proceedings : Annual International Conference of the IEEE Engineering in Medicine and Biology Society IEEE Engineering in Medicine and Biology Society Conference. 2007;2007:1354-7. doi:10.1109/IEMBS.2007.4352549.

40. Yokoe M, Okuno R, Hamasaki T, Kurachi Y, Akazawa K, Sakoda S. Opening velocity, a novel parameter, for finger tapping test in patients with Parkinson's disease. Parkinsonism & related disorders. 2009;15:440-4. doi:10.1016/j.parkreldis.2008.11.003.

41. Stamatakis J, Ambroise J, Crémers J, Sharei H, Delvaux V, Macq B et al. Finger tapping clinimetric score prediction in Parkinson's disease using low-cost accelerometers. Computational intelligence and neuroscience. 2013;2013:717853. doi:10.1155/2013/717853.

42. Chastin SF, Baker K, Jones D, Burn D, Granat MH, Rochester L. The pattern of habitual sedentary behavior is different in advanced Parkinson's disease. Mov Disord. 2010;25(13):2114-20. doi:10.1002/mds.23146.

43. Wile DJ, Ranawaya R, Kiss ZH. Smart watch accelerometry for analysis and diagnosis of tremor. J Neurosci Methods. 2014;230:1-4. doi:10.1016/j.jneumeth.2014.04.021.

44. Moore ST, MacDougall HG, Gracies JM, Cohen HS, Ondo WG. Long-term monitoring of gait in Parkinson's disease. Gait Posture. 2007;26(2):200-7. doi:S0966-6362(06)00190-1

45. Moore ST, MacDougall HG, Ondo WG. Ambulatory monitoring of freezing of gait in Parkinson's disease. J Neurosci Methods. 2008;167(2):340-8. doi:S0165-0270(07)00428-1

46. Nombela C, Pedreno-Molina JL, Ros-Bernal F, Molina-Vilaplana J, Fdez-Villalba E, Lopez-Coronado J et al. Dopamine modulation affects the performance of parkinsonian patients in a precision motor task measured by an antropomorphic device. Hum Mov Sci. 2012;31(3):730-42. doi:S0167-9457(11)00118-7

47. Mitoma H, Yoneyama M, Orimo S. 24-hour recording of parkinsonian gait using a portable gait rhythmogram. Intern Med. 2010;49(22):2401-8.

48. Han J, Jeon HS, Yi WJ, Jeon BS, Park KS. Adaptive windowing for gait phase discrimination in Parkinsonian gait using 3-axis acceleration signals. Med Biol Eng Comput. 2009;47(11):1155-64. doi:10.1007/s11517-009-0521-5.

49. Caligiuri MP, Tripp RM. A portable hand-held device for quantifying and standardizing tremor assessment. Journal of medical engineering & technology.28:254-62. doi:10.1080/03091900410001658111.

50. Esculier J-F, Vaudrin J, Bériault P, Gagnon K, Tremblay LE. Home-based balance training programme using Wii Fit with balance board for Parkinsons's disease: a pilot study. Journal of rehabilitation medicine : official journal of the UEMS European Board of Physical and Rehabilitation Medicine. 2012;44:144-50. doi:10.2340/16501977-0922.

51. Pompeu JE, Mendes FADS, Silva KGd, Lobo AM, Oliveira TdP, Zomignani AP et al. Effect of Nintendo Wii™-based motor and cognitive training on activities of daily living in patients with Parkinson's disease: a randomised clinical trial. Physiotherapy. 2012;98:196-204. doi:10.1016/j.physio.2012.06.004.

52. Clark RA, Bryant AL, Pua Y, McCrory P, Bennell K, Hunt M. Validity and reliability of the Nintendo Wii Balance Board for assessment of standing balance. Gait Posture. 2010;31(3):307-10. doi:S0966-6362(09)00664-X

53. Chien SL, Lin SZ, Liang CC, Soong YS, Lin SH, Hsin YL et al. The efficacy of quantitative gait analysis by the GAITRite system in evaluation of parkinsonian bradykinesia. Parkinsonism Relat Disord. 2006;12(7):438-42. doi:S1353-8020(06)00087-3

54. Menz HB, Latt MD, Tiedemann A, Mun San Kwan M, Lord SR. Reliability of the GAITRite walkway system for the quantification of temporo-spatial parameters of gait in young and older people. Gait & posture. 2004;20:20-5. doi:10.1016/S0966-6362(03)00068-7.

55. Brach JS, Perera S, Studenski S, Katz M, Hall C, Verghese J. Meaningful change in measures of gait variability in older adults. Gait & posture. 2010;31:175-9. doi:10.1016/j.gaitpost.2009.10.002.

56. Nantel J, de Solages C, Bronte-Stewart H. Repetitive stepping in place identifies and measures freezing episodes in subjects with Parkinson's disease. Gait & posture. 2011;34:329-33. doi:10.1016/j.gaitpost.2011.05.020.

57. Termoz N, Halliday SE, Winter DA, Frank JS, Patla AE, Prince F. The control of upright stance in young, elderly and persons with Parkinson's disease. Gait & posture. 2008;27:463-70. doi:10.1016/j.gaitpost.2007.05.015.

58. Rocchi L, Chiari L, Horak FB. Effects of deep brain stimulation and levodopa on postural sway in Parkinson's disease. J Neurol Neurosurg Psychiatry. 2002;73(3):267-74.

59. Corriveau H, Hebert R, Prince F, Raiche M. Postural control in the elderly: an analysis of test-retest and interrater reliability of the COP-COM variable. Arch Phys Med Rehabil. 2001;82(1):80-5. doi:S0003-9993(01)53407-3

60. Frenklach A, Louie S, Koop MM, Bronte-Stewart H. Excessive postural sway and the risk of falls at different stages of Parkinson's disease. Mov Disord. 2009;24(3):377-85. doi:10.1002/mds.22358.

61. Kara B, Genc A, Colakoglu BD, Cakmur R. The effect of supervised exercises on static and dynamic balance in Parkinson's disease patients. NeuroRehabilitation. 2012;30:351-7. doi:10.3233/NRE-2012-0766.

62. Liston RA, Brouwer BJ. Reliability and validity of measures obtained from stroke patients using the Balance Master. Arch Phys Med Rehabil. 1996;77(5):425-30. doi:S0003-9993(96)90028-3

63. Pickerill ML, Harter RA. Validity and reliability of limits-of-stability testing: a comparison of 2 postural stability evaluation devices. Journal of athletic training.46:600-6.

64. Viitasalo MK, Kampman V, Sotaniemi KA, Leppavuori S, Myllyla VV, Korpelainen JT. Analysis of sway in Parkinson's disease using a new inclinometry-based method. Mov Disord. 2002;17(4):663-9. doi:10.1002/mds.10023.

65. Matinolli M, Korpelainen JT, Korpelainen R, Sotaniemi KA, Virranniemi M, Myllyla VV. Postural sway and falls in Parkinson's disease: a regression approach. Mov Disord. 2007;22(13):1927-35. doi:10.1002/mds.21633.

66. Paalanne NP, Korpelainen R, Taimela SP, Remes J, Salakka M, Karppinen JI. Reproducibility and reference values of inclinometric balance and isometric trunk muscle strength measurements in Finnish young adults. Journal of strength and conditioning research / National Strength & Conditioning Association. 2009;23:1618-26.

67. Blaszczyk JW, Orawiec R, Duda-Klodowska D, Opala G. Assessment of postural instability in patients with Parkinson's disease. Exp Brain Res. 2007;183(1):107-14. doi:10.1007/s00221-007-1024-y.

68. Blaszczyk JW, Orawiec R. Assessment of postural control in patients with Parkinson's disease: sway ratio analysis. Hum Mov Sci. 2011;30(2):396-404.

69. Murray JF. Construction of a stabilometer capable of indicating the variability of non-level performance. Perceptual and motor skills. 1982;55:1211-5.

70. Chiviacowsky S, Wulf G, Wally R. An external focus of attention enhances balance learning in older adults. Gait Posture. 2010;32(4):572-5. doi:S0966-6362(10)00228-6

71. Chiviacowsky S, Wulf G, Lewthwaite R, Campos T. Motor learning benefits of self-controlled practice in persons with Parkinson's disease. Gait Posture. 2012;35(4):601-5. doi:S0966-6362(11)00802-2

72. Caligiuri MP, Teulings H-L, Filoteo JV, Song D, Lohr JB. Quantitative measurement of handwriting in the assessment of drug-induced parkinsonism. Human movement science. 2006;25:510-22. doi:10.1016/j.humov.2006.02.004.

73. Saunders-Pullman R, Derby C, Stanley K, Floyd A, Bressman S, Lipton RB et al. Validity of spiral analysis in early Parkinson's disease. Movement disorders: official journal of the Movement Disorder Society. 2008;23:531-7. doi:10.1002/mds.21874.

74. Wang S, Bain PG, Aziz TZ, Liu X. The direction of oscillation in spiral drawings can be used to differentiate distal and proximal arm tremor. Neuroscience letters.384:188-92. doi:10.1016/j.neulet.2005.04.084.

75. Liu X, Carroll CB, Wang S-Y, Zajicek J, Bain PG. Quantifying drug-induced dyskinesias in the arms using digitised spiral-drawing tasks. Journal of neuroscience methods. 2005;144:47-52. doi:10.1016/j.jneumeth.2004.10.005.

76. Eichhorn TE, Gasser T, Mai N, Marquardt C, Arnold G, Schwarz J et al. Computational analysis of open loop handwriting movements in Parkinson's disease: a rapid method to detect dopamimetic effects. Movement disorders: official journal of the Movement Disorder Society. 1996;11:289-97. doi:10.1002/mds.870110313.

77. Bronte-Stewart HM, Ding L, Alexander C, Zhou Y, Moore GP. Quantitative digitography (QDG): a sensitive measure of digital motor control in idiopathic Parkinson's disease. Movement disorders: official journal of the Movement Disorder Society. 2000;15:36-47.

78. Lou J-S, Kearns G, Benice T, Oken B, Sexton G, Nutt J. Levodopa improves physical fatigue in Parkinson's disease: a double-blind, placebo-controlled, crossover study. Movement disorders : official journal of the Movement Disorder Society. 2003;18:1108-14. doi:10.1002/mds.10505.

79. Jabusch H-C, Vauth H, Altenmüller E. Quantification of focal dystonia in pianists using scale analysis. Movement disorders: official journal of the Movement Disorder Society. 2004;19:171-80. doi:10.1002/mds.10671.

80. Taylor Tavares AL, Jefferis GSXE, Koop M, Hill BC, Hastie T, Heit G et al. Quantitative measurements of alternating finger tapping in Parkinson's disease correlate with UPDRS motor disability and reveal the improvement in fine motor control from medication and deep brain stimulation. Movement disorders: official journal of the Movement Disorder Society. 2005;20:1286-98. doi:10.1002/mds.20556.

81. Koop MM, Shivitz N, Brontë-Stewart H. Quantitative measures of fine motor, limb, and postural bradykinesia in very early stage, untreated Parkinson's disease. Movement disorders : official journal of the Movement Disorder Society. 2008;23:1262-8. doi:10.1002/mds.22077.

82. Mirek E, Rudzinska M, Szczudlik A. The assessment of gait disorders in patients with Parkinson's disease using the three-dimensional motion analysis system Vicon. Neurol Neurochir Pol. 2007;41(2):128-33.

83. Delval A, Salleron J, Bourriez JL, Bleuse S, Moreau C, Krystkowiak P et al. Kinematic angular parameters in PD: reliability of joint angle curves and comparison with healthy subjects. Gait Posture. 2008;28(3):495-501. doi:S0966-6362(08)00068-4

84. Delval A, Snijders AH, Weerdesteyn V, Duysens JE, Defebvre L, Giladi N et al. Objective detection of subtle freezing of gait episodes in Parkinson's disease. Movement disorders : official journal of the Movement Disorder Society. 2010;25:1684-93. doi:10.1002/mds.23159.

85. Das S, Trutoiu L, Murai A, Alcindor D, Oh M, De la Torre F et al. Quantitative measurement of motor symptoms in Parkinson's disease: a study with full-body motion capture data. Conference proceedings: Annual International Conference of the IEEE Engineering in Medicine and Biology Society IEEE Engineering in Medicine and Biology Society Conference. 2011;2011:6789-92. doi:10.1109/IEMBS.2011.6091674.

86. Mesure S, Azulay JP, Pouget J, Amblard B. Strategies of segmental stabilization during gait in Parkinson's disease. Experimental brain research Experimentelle Hirnforschung Expérimentation cérébrale. 1999;129:573-81.

87. Bennett KM, O'Sullivan JD, Peppard RF, McNeill PM, Castiello U. The effect of unilateral posteroventral pallidotomy on the kinematics of the reach to grasp movement. Journal of neurology, neurosurgery, and psychiatry. 1998;65:479-87.

88. Agostino R, Currà A, Giovannelli M, Modugno N, Manfredi M, Berardelli A. Impairment of individual finger movements in Parkinson's disease. Movement disorders: official journal of the Movement Disorder Society. 2003;18:560-5. doi:10.1002/mds.10313.

89. Rao AS, Bodenheimer RE, Davis TL, Li R, Voight C, Dawant BM. Quantifying drug induced dyskinesia in Parkinson's disease patients using standardized videos. Conference proceedings: Annual International Conference of the IEEE Engineering in Medicine and Biology Society IEEE Engineering in Medicine and Biology Society Conference. 2008;2008:1769-72. doi:10.1109/IEMBS.2008.4649520.

90. Rao AS, Dawant BM, Bodenheimer RE, Li R, Fang J, Phibbs F et al. Validating an objective video-based dyskinesia severity score in Parkinson's disease patients. Parkinsonism & related disorders. 2013;19:232-7. doi:10.1016/j.parkreldis.2012.10.015.

91. Ferrarin M, Carpinella I, Rabuffetti M, Calabrese E, Mazzoleni P, Nemni R. Locomotor disorders in patients at early stages of Parkinson's disease: a quantitative analysis. Conf Proc IEEE Eng Med Biol Soc. 2006;1:1224-7. doi:10.1109/IEMBS.2006.260677.

92. Zhang T, Wei G, Yan Z, Ding M, Li C, Ding H et al. Quantitative assessment of Parkinson's disease deficits. Chinese medical journal. 1999;112:812-5.

93. Cho C, Osaki Y, Kunin M, Cohen B, Olanow CW, Raphan T. A model-based approach for assessing parkinsonian gait and effects of levodopa and deep brain stimulation. Conference proceedings : Annual International Conference of the IEEE Engineering in Medicine and Biology Society IEEE Engineering in Medicine and Biology Society Conference. 2006;1:1228-31. doi:10.1109/IEMBS.2006.259439.

94. Jobbágy A, Harcos P, Karoly R, Fazekas G. Analysis of finger-tapping movement. Journal of neuroscience methods. 2005;141:29-39. doi:10.1016/j.jneumeth.2004.05.009.

95. Joebges M, Mrowka M, Schimke N, Shing M, Dengler R, Odin P. Three-dimensional computerized analysis of diadochokinetic movements of Parkinsonian patients. Acta neurologica Scandinavica. 2003;108:415-23.

96. Bäzner H, Schanz J, Blahak C, Grips E, Wöhrle JC, Hennerici M. Differential pattern of hand-tapping compromise in vascular versus idiopathic parkinsonism: a study based on computerized movement analysis. Movement disorders : official journal of the Movement Disorder Society. 2005;20:504-8. doi:10.1002/mds.20372.

97. Timmermann L, Braun M, Groiss S, Wojtecki L, Ostrowski S, Krause H et al. Differential effects of levodopa and subthalamic nucleus deep brain stimulation on bradykinesia in Parkinson's disease. Movement disorders : official journal of the Movement Disorder Society. 2008;23:218-27. doi:10.1002/mds.21808.

98. Hermsdörfer J, Marquardt C, Wack S, Mai N. Comparative analysis of diadochokinetic movements. Journal of electromyography and kinesiology : official journal of the International Society of Electrophysiological Kinesiology. 1999;9:283-95.

99. Van Wieringen M, Eklund J. Real-time signal processing of accelerometer data for wearable medical patient monitoring devices. Conference proceedings : Annual International Conference of the IEEE Engineering in Medicine and Biology Society IEEE Engineering in Medicine and Biology Society Conference. 2008;2008:2397-400. doi:10.1109/IEMBS.2008.4649682.

100. Mamorita N, Iizuka T, Takeuchi A, Shirataka M, Ikeda N. Development of a system for measurement and analysis of tremor using a three-axis accelerometer. Methods of information in medicine. 2009;48:589-94. doi:10.3414/ME9243.

101. Synnott J, Chen L, Nugent CD, Moore G. WiiPD--an approach for the objective home assessment of Parkinson's disease. Conference proceedings : Annual International Conference of the IEEE Engineering in Medicine and Biology Society IEEE Engineering in Medicine and Biology Society Conference. 2011;2011:2388-91. doi:10.1109/IEMBS.2011.6090666.

102. Synnott J, Chen L, Nugent CD, Moore G. WiiPD--objective home assessment of Parkinson's disease using the Nintendo Wii remote. IEEE transactions on information technology in biomedicine : a publication of the IEEE Engineering in Medicine and Biology Society. 2012;16:1304-12. doi:10.1109/TITB.2012.2215878.

103. Sauermann S, Standhardt H, Gerschlager W, Lanmüller H, Alesch F. Kinematic evaluation in Parkinson's disease using a hand-held position transducer and computerized signal analysis. Acta neurochirurgica. 2005;147:939-45; discussion 45. doi:10.1007/s00701-005-0569-4.

104. Allen DP, Playfer JR, Aly NM, Duffey P, Heald A, Smith SL et al. On the use of low-cost computer peripherals for the assessment of motor dysfunction in Parkinson's disease--quantification of bradykinesia using target tracking tasks. IEEE transactions on neural systems and rehabilitation engineering : a publication of the IEEE Engineering in Medicine and Biology Society. 2007;15:286-94. doi:10.1109/TNSRE.2007.897020.

105. Kraus PH, Hoffmann A. Spiralometry: computerized assessment of tremor amplitude on the basis of spiral drawing. Movement disorders : official journal of the Movement Disorder Society. 2010;25:2164-70. doi:10.1002/mds.23193.

106. Giovannoni G, van Schalkwyk J, Fritz VU, Lees AJ. Bradykinesia akinesia inco-ordination test (BRAIN TEST): an objective computerised assessment of upper limb motor function. Journal of neurology, neurosurgery, and psychiatry. 1999;67:624-9.

107. Homann CN, Suppan K, Wenzel K, Giovannoni G, Ivanic G, Horner S et al. The Bradykinesia Akinesia Incoordination Test (BRAIN TEST), an objective and user-friendly means to evaluate patients with parkinsonism. Movement disorders : official journal of the Movement Disorder Society. 2000;15:641-7.

108. Kondraske GV, Stewart RM. Web-based evaluation of Parkinson's disease subjects: objective performance capacity measurements and subjective characterization profiles. Conference proceedings : Annual International Conference of the IEEE Engineering in Medicine and Biology Society IEEE Engineering in Medicine and Biology Society Conference. 2008;2008:799-802. doi:10.1109/IEMBS.2008.4649273.

109. Cunningham L, Mason S, Nugent C, Moore G, Finlay D, Craig D. Home-based monitoring and assessment of Parkinson's disease. IEEE transactions on information technology in biomedicine : a publication of the IEEE Engineering in Medicine and Biology Society. 2011;15:47-53. doi:10.1109/TITB.2010.2091142.

110. Cunningham LM, Nugent CD, Moore G, Finlay DD, Craig D. Computer-based assessment of movement difficulties in Parkinson's disease. Computer methods in biomechanics and biomedical engineering. 2012;15:1081-92. doi:10.1080/10255842.2011.571678.

111. Skodda S, Visser W, Schlegel U. Gender-related patterns of dysprosody in Parkinson disease and correlation between speech variables and motor symptoms. Journal of voice: official journal of the Voice Foundation. 2011;25:76-82. doi:10.1016/j.jvoice.2009.07.005.

112. Liss JM, LeGendre S, Lotto AJ. Discriminating dysarthria type from envelope modulation spectra. Journal of speech, language, and hearing research: JSLHR. 2010;53:1246-55. doi:10.1044/1092-4388(2010/09-0121).

113. Little MA, McSharry PE, Hunter EJ, Spielman J, Ramig LO. Suitability of dysphonia measurements for telemonitoring of Parkinson's disease. IEEE transactions on bio-medical engineering. 2009;56:1015. doi:10.1109/TBME.2008.2005954.

114. Shao J, MacCallum JK, Zhang Y, Sprecher A, Jiang JJ. Acoustic analysis of the tremulous voice: assessing the utility of the correlation dimension and perturbation parameters. Journal of communication disorders.43:35-44. doi:10.1016/j.jcomdis.2009.09.001.

115. Goberman AM, Elmer LW. Acoustic analysis of clear versus conversational speech in individuals with Parkinson disease. Journal of communication disorders.38:215-30. doi:10.1016/j.jcomdis.2004.10.001.

116. Marks L, Weinreich J. Drooling in Parkinson's disease: a novel tool for assessment of swallow frequency. International journal of language & communication disorders / Royal College of Speech & Language Therapists. 2001;36 Suppl:288-91.

117. Lee G-S, Lin S-H. Changes of rhythm of vocal fundamental frequency in sensorineural hearing loss and in Parkinson's disease. The Chinese journal of physiology. 2009;52:446-50.

118. Nogaki H, Fukusako T, Sasabe F, Negoro K, Morimatsu M. Muscle strength in early Parkinson's disease. Movement disorders: official journal of the Movement Disorder Society. 1995;10:225-6. doi:10.1002/mds.870100218.

119. Nogaki H, Kakinuma S, Morimatsu M. Movement velocity dependent muscle strength in Parkinson's disease. Acta neurologica Scandinavica. 1999;99:152-7.

120. Bohannon RW. Variability and reliability of the pendulum test for spasticity using a Cybex II isokinetic dynamometer. Physical therapy. 1987;67:659-61.

121. Molczyk L, Thigpen LK, Eickhoff J, Goldgar D, Gallagher JC. Reliability of Testing the Knee Extensors and Flexors in Healthy Adult Women Using a Cybex II Isokinetic Dynamometer. The Journal of orthopaedic and sports physical therapy. 1991;14:37-41. doi:10.2519/jospt.1991.14.1.37.

122. Barlow SM, Abbs JH. Force transducers for the evaluation of labial, lingual, and mandibular motor impairments. Journal of speech and hearing research. 1983;26:616-21.

123. Wood LM, Hughes J, Hayes KC, Wolfe DL. Reliability of labial closure force measurements in normal subjects and patients with CNS disorders. Journal of speech and hearing research. 1992;35:252-8.

124. Sepehri B, Esteki A, Ebrahimi-Takamjani E, Shahidi GA, Khamseh F, Moinodin M. Quantification of rigidity in Parkinson's disease. Ann Biomed Eng. 2007;35(12):2196-203. doi:10.1007/s10439-007-9379-6.

125. Baziyan BK, Chigaleichik LA, Teslenko EL, Damyanovich EV, Poleshuk VV, Swetsov AY et al. Hardware-program complex for inspection of eyes, head and hand movements coordination of the man. Conference proceedings : Annual International Conference of the IEEE Engineering in Medicine and Biology Society IEEE Engineering in Medicine and Biology Society Conference. 2008;2008:1773-6. doi:10.1109/IEMBS.2008.4649521.

126. Wong MN, Murdoch BE, Whelan B-M. Kinematic analysis of lingual function in dysarthric speakers with Parkinson's disease: An electromagnetic articulograph study. International journal of speech-language pathology. 2010;12:414-25. doi:10.3109/17549507.2010.495784.

127. Wong MN, Murdoch BE, Whelan B-M. Lingual kinematics during rapid syllable repetition in Parkinson's disease. International journal of language & communication disorders / Royal College of Speech & Language Therapists.47:578-88. doi:10.1111/j.1460-6984.2012.00167.

128. Kandori A, Yokoe M, Sakoda S, Abe K, Miyashita T, Oe H et al. Quantitative magnetic detection of finger movements in patients with Parkinson's disease. Neuroscience research. 2004;49:253-60. doi:10.1016/j.neures.2004.03.004.

129. Sano Y, Kandori A, Shima K, Tamura Y, Takagi H, Tsuji T et al. Reliability of Finger Tapping Test Used in Diagnosis of Movement Disorders: IEEE; 2011.

130. Sano Y, Kandori A, Miyoshi T, Tsuji T, Shima K, Yokoe M et al. Severity estimation of finger-tapping caused by Parkinson's disease by using linear discriminant regression analysis. Conference proceedings : Annual International Conference of the IEEE Engineering in Medicine and Biology Society IEEE Engineering in Medicine and Biology Society Conference. 2012;2012:4315-8. doi:10.1109/EMBC.2012.6346921.

131. Zhang J. Basic gait analysis based on continuous wave radar. Gait & posture. 2012;36:667-71. doi:10.1016/j.gaitpost.2012.04.020.

132. Giuffrida JP, Riley DE, Maddux BN, Heldman DA. Clinically deployable Kinesia technology for automated tremor assessment. Mov Disord. 2009;24(5):723-30. doi:10.1002/mds.22445.

133. Patel S, Chen BR, Buckley T, Rednic R, McClure D, Tarsy D et al. Home monitoring of patients with Parkinson's disease via wearable technology and a web-based application. Conf Proc IEEE Eng Med Biol Soc. 2010;2010:4411-4. doi:10.1109/IEMBS.2010.5627124.

134. Chen BR, Patel S, Buckley T, Rednic R, McClure DJ, Shih L et al. A web-based system for home monitoring of patients with Parkinson's disease using wearable sensors. IEEE Trans Biomed Eng. 2010;58(3):831-6. doi:10.1109/TBME.2010.2090044.

135. Chen BR, Patel S, Buckley T, Rednic R, McClure DJ, Shih L et al. A web-based system for home monitoring of patients with Parkinson's disease using wearable sensors. IEEE Trans Biomed Eng. 2011;58(3):831-6. doi:10.1109/TBME.2010.2090044.

136. Patel S, Chen BR, Mancinelli C, Paganoni S, Shih L, Welsh M et al. Longitudinal monitoring of patients with Parkinson's disease via wearable sensor technology in the home setting. Conf Proc IEEE Eng Med Biol Soc. 2011;2011:1552-5. doi:10.1109/IEMBS.2011.6090452.

137. Barth J, Klucken J, Kugler P, Kammerer T, Steidl R, Winkler J et al. Biometric and mobile gait analysis for early diagnosis and therapy monitoring in Parkinson's disease. Conf Proc IEEE Eng Med Biol Soc. 2011;2011:868-71. doi:10.1109/IEMBS.2011.6090226.

138. Piro NE, Baumann L, Tengler M, Piro L, Blechschmidt-Trapp R. Telemonitoring of patients with Parkinson's disease using inertia sensors. Applied clinical informatics. 2014;5(2):503-11. doi:10.4338/ACI-2014-04-RA-0046.

139. Cancela J, Pansera M, Arredondo MT, Estrada JJ, Pastorino M, Pastor-Sanz L et al. A comprehensive motor symptom monitoring and management system: the bradykinesia case. Conf Proc IEEE Eng Med Biol Soc. 2010;2010:1008-11. doi:10.1109/IEMBS.2010.5627775.

140. Cancela J, Pastorino M, Arredondo MT, Pansera M, Pastor-Sanz L, Villagra F et al. Gait assessment in Parkinson's disease patients through a network of wearable accelerometers in unsupervised environments. Conf Proc IEEE Eng Med Biol Soc. 2011;2011:2233-6. doi:10.1109/IEMBS.2011.6090423.

141. Pastorino M, Cancela J, Arredondo MT, Pansera M, Pastor-Sanz L, Villagra F et al. Assessment of Bradykinesia in Parkinson's disease patients through a multi-parametric system. Conf Proc IEEE Eng Med Biol Soc. 2011;2011:1810-3. doi:10.1109/IEMBS.2011.6090516.

142. Tzallas AT, Tsipouras MG, Rigas G, Tsalikakis DG, Karvounis EC, Chondrogiorgi M et al. PERFORM: a system for monitoring, assessment and management of patients with Parkinson's disease. Sensors (Basel). 2014;14(11):21329-57. doi:10.3390/s141121329.

143. Cancela J, Pastorino M, Tzallas AT, Tsipouras MG, Rigas G, Arredondo MT et al. Wearability assessment of a wearable system for Parkinson's disease remote monitoring based on a body area network of sensors. Sensors (Basel). 2014;14(9):17235-55. doi:10.3390/s140917235.

144. Goetz CG, Stebbins GT, Wolff D, DeLeeuw W, Bronte-Stewart H, Elble R et al. Testing objective measures of motor impairment in early Parkinson's disease: Feasibility study of an at-home testing device. Mov Disord. 2009;24(4):551-6. doi:10.1002/mds.22379.

145. Tsanas A, Little MA, McSharry PE, Ramig LO. Accurate telemonitoring of Parkinson's disease progression by noninvasive speech tests. IEEE transactions on bio-medical engineering. 2010;57:884-93. doi:10.1109/TBME.2009.2036000.

146. Barroso Junior MC, Esteves GP, Nunes TP, Silva LM, Faria AC, Melo PL. A telemedicine instrument for remote evaluation of tremor: design and initial applications in fatigue and patients with Parkinson's disease. Biomed Eng Online. 2011;10:14. doi:1475-925X-10-14

147. Ying H, Schlosser M, Schnitzer A, Schafer T, Schlafke ME, Leonhardt S et al. Distributed intelligent sensor network for the rehabilitation of Parkinson's patients. IEEE Trans Inf Technol Biomed. 2011;15(2):268-76. doi:10.1109/TITB.2010.2095463.

148. Rissanen SM, Kankaanpaa M, Tarvainen MP, Nuutinen J, Airaksinen O, Karjalainen PA. EMG and acceleration signal analysis for quantifying the effects of medication in Parkinson's disease. Conference proceedings : Annual International Conference of the IEEE Engineering in Medicine and Biology Society IEEE Engineering in Medicine and Biology Society Conference. 2011;2011:7496-9. doi:10.1109/IEMBS.2011.6091848.

149. Meigal AY, Rissanen SM, Tarvainen MP, Georgiadis SD, Karjalainen PA, Airaksinen O et al. Linear and nonlinear tremor acceleration characteristics in patients with Parkinson's disease. Physiological measurement. 2012;33:395-412. doi:10.1088/0967-3334/33/3/395.

150. Cole BT, Roy SH, Nawab SH. Detecting freezing-of-gait during unscripted and unconstrained activity. Conference proceedings : Annual International Conference of the IEEE Engineering in Medicine and Biology Society IEEE Engineering in Medicine and Biology Society Conference. 2011;2011:5649-52. doi:10.1109/IEMBS.2011.6091367.

151. Roy SH, Cole BT, Gilmore LD, De Luca CJ, Nawab SH. Resolving signal complexities for ambulatory monitoring of motor function in Parkinson's disease. Conference proceedings : Annual International Conference of the IEEE Engineering in Medicine and Biology Society IEEE Engineering in Medicine and Biology Society Conference. 2011;2011:4836-9. doi:10.1109/IEMBS.2011.6091198.

152. Beuter A, de Geoffroy A, Edwards R. Analysis of rapid alternating movements in Cree subjects exposed to methylmercury and in subjects with neurological deficits. Environ Res. 1999;80(1):64-79. doi:S0013-9351(98)93885-3

153. Fimbel EJ, Domingo PP, Lamoureux D, Beuter A. Automatic detection of movement disorders using recordings of rapid alternating movements. Journal of neuroscience methods. 2005;146:183-90. doi:10.1016/j.jneumeth.2005.02.007.

154. Niwa F, Kuriyama N, Nakagawa M, Imanishi J. Circadian rhythm of rest activity and autonomic nervous system activity at different stages in Parkinson's disease. Autonomic neuroscience : basic & clinical. 2011;165:195-200. doi:10.1016/j.autneu.2011.07.010.

155. Conradsson D, Lofgren N, Stahle A, Hagstromer M, Franzen E. A novel conceptual framework for balance training in Parkinson's disease-study protocol for a randomised controlled trial. BMC Neurol. 2012;12:111. doi:10.1186/1471-2377-12-111.

156. Sasaki JE, John D, Freedson PS. Validation and comparison of ActiGraph activity monitors. Journal of science and medicine in sport / Sports Medicine Australia. 2011;14(5):411-6. doi:10.1016/j.jsams.2011.04.003.

157. Bächlin M, Plotnik M, Roggen D, Maidan I, Hausdorff JM, Giladi N et al. Wearable assistant for Parkinson's disease patients with the freezing of gait symptom. IEEE transactions on information technology in biomedicine : a publication of the IEEE Engineering in Medicine and Biology Society. 2010;14:436-46. doi:10.1109/TITB.2009.2036165.

158. Askari S, Zhang M, Won DS. An EMG-based system for continuous monitoring of clinical efficacy of Parkinson's disease treatments. Conference proceedings : Annual International Conference of the IEEE Engineering in Medicine and Biology Society IEEE Engineering in Medicine and Biology Society Conference. 2010;2010:98-101. doi:10.1109/IEMBS.2010.5626133.

159. Popovic MB, Djuric-Jovicic M, Radovanovic S, Petrovic I, Kostic V. A simple method to assess freezing of gait in Parkinson's disease patients. Braz J Med Biol Res. 2010;43(9):883-9. doi:S0100-879X2010007500077.
